# Influenza Vaccination among Underserved African-American Older Adults

**DOI:** 10.1155/2020/2160894

**Published:** 2020-11-05

**Authors:** Mohsen Bazargan, Cheryl Wisseh, Edward Adinkrah, Hoorolnesa Ameli, Delia Santana, Sharon Cobb, Shervin Assari

**Affiliations:** ^1^Department of Family Medicine, Charles R. Drew University of Medicine and Science (CDU), Los Angeles, California, USA; ^2^Department of Public Health, CDU, Los Angeles, California, USA; ^3^Physician Assistant Program, CDU, Los Angeles, California, USA; ^4^Department of Family Medicine, UCLA, Los Angeles, California, USA; ^5^Department of Clinical Pharmacy Practice, School of Pharmacy and Pharmaceutical Sciences, University of California at Irvine, California, USA; ^6^Department of Emergency Medicine, Qom University of Medical Sciences, Gom, Iran; ^7^School of Nursing, CDU, Los Angeles, California, USA

## Abstract

**Background:**

Racial disparities in influenza vaccination among underserved minority older adults are a public health problem. Understanding the factors that impact influenza vaccination behaviors among underserved older African-Americans could lead to more effective communication and delivery strategies.

**Aims:**

We aimed to investigate rate and factors associated with seasonal influenza vaccination among underserved African-American older adults. We were particularly interested in the roles of demographic factors, socioeconomic status, and continuity and patient satisfaction with medical care, as well as physical and mental health status.

**Methods:**

This community-based cross-sectional study recruited 620 African-American older adults residing in South Los Angeles, one of the most under-resources areas within Los Angeles County, with a population of over one million. Bivariate and multiple regression analyses were performed to document independent correlates of influenza vaccination.

**Results:**

One out of three underserved African-American older adults aged 65 years and older residing in South Los Angeles had never been vaccinated against the influenza. Only 49% of participants reported being vaccinated within the 12 months prior to the interview. One out of five participants admitted that their health care provider recommended influenza vaccination. However, only 45% followed their provider's recommendations. Multivariate logistic regression shows that old-old (≥75 years), participants who lived alone, those with a lower level of continuity of care and satisfaction with the accessibility, availability, and quality of care, and participants with a higher number of depression symptoms were less likely to be vaccinated. As expected, participants who indicated that their physician had advised them to obtain a flu vaccination were more likely to be vaccinated. Our data shows that only gender was associated with self-report of being advised to have a flu shot. *Discussion*. One of the most striking aspects of this study is that no association between influenza vaccination and being diagnosed with chronic obstructive pulmonary disease or other major chronic condition was detected. Our study confirmed that both continuity of care and satisfaction with access, availability, and quality of medical care are strongly associated with current influenza vaccinations. We documented that participants with a higher number of depression symptoms were less likely to be vaccinated.

**Conclusion:**

These findings highlight the role that culturally acceptable and accessible usual source of care van play as a gatekeeper to facilitate and implement flu vaccination among underserved minority older adults. Consistent disparities in influenza vaccine uptake among underserved African-American older adults, coupled with a disproportionate burden of chronic diseases, places them at high risk for undesired outcomes associated with influenza. As depression is more chronic/disabling and is less likely to be treated in African-Americans, there is a need to screen and treat depression as a strategy to enhance preventive care management such as vaccination of underserved African-American older adults. Quantification of associations between lower vaccine uptake and both depression symptoms as well as living alone should enable health professionals target underserved African-American older adults who are isolated and suffer from depression to reduce vaccine-related inequalities.

## 1. Introduction

In today's environment of emerging diseases, COVID-19 has brought on a new priority in the need for influenza vaccination among older adults [1–3]. COVID-19 is disproportionally affecting not broadly older adults, but older African-Americans two times more than older Caucasians [4]. Such a realization is important enough for health care providers to place new emphasis on the urgent need to increase influenza vaccination among older African-Americans. Indeed, race and age are among the most consistent sociodemographic predictors of influenza vaccination [5–9]. Literature on racial disparities in vaccine administration has evidenced lower influenza vaccination rates of African-American older adults compared to their White counterparts despite having higher rates of chronic diseases [10]. These low rates have been associated with concerns around social norms, lack of trust, insufficient influenza education, and belief systems [11–15]. Within major urban areas, such as Los Angeles and New York, the influenza vaccination rate is even lower among African-American than among Hispanic adults aged 50 or older [16]. The low rates of influenza vaccination rates among older African-Americans present a major public health challenge [5, 17, 18]. One study found that the proportion of individuals that are at an increased risk of influenza complications because of comorbid health conditions varied 10-folds by age. Furthermore, the age-specific prevalence of multimorbidity is also highest in African-Americans of all age groups. Thus, understanding the factors that impact influenza vaccination behaviors among African-Americans could lead to more effective strategies to increase vaccination rate and enhance delivery strategies [19].

There is a considerable need to explore differences related to the influenza vaccine rate within African-American communities. Evidence has shown that African-Americans have lower knowledge levels of the flu vaccine in addition to heightened hesitancy and decreased trust toward the vaccine and the vaccination process [20, 21]. The weak relationship of trusting the flu vaccine may be attributed to a lack of strong recommendation of the vaccine by health care providers for African-Americans [22]. In addition, older African-Americans are likely to disbelieve the preventative ability of the vaccine or experience adverse side effects [23]. A recent study documented the considerable diversity within the African-American population related to influenza vaccination decisions and highlighted the need to “break down the monolith” in future research by examining social, behavioral, and health differences [24]. This is particularly important because African-American communities are not comparable in terms of economic conditions, and despite the growth of the African-American middle class, a majority of the African-American community continues to reside in under-resourced areas. This can increase the risk of influenza being a threatening and potentially deadly illness for this group of older adults [23].

The purpose of this study was to investigate seasonal influenza vaccination rates among older African-American adults residing in South Los Angeles. Specifically, we aimed to examine correlates associated with influenza vaccination, with a particular focus on continuity and patient satisfaction with medical care and physical and mental health status. This is important as the target population includes underserved older African-American adults living in a critically under-resourced service area of Los Angeles County.

## 2. Materials and Methods

This cross-sectional survey was conducted between 2015 and 2018 among older African-American residing in South Los Angeles. More than one million Americans live in Service Planning Area 6 (SPA 6), located in South Central Los Angeles. Compared to the rest of the Los Angeles County, SPA 6 residents are disproportionately affected by health disparities [25].

Data was collected via structured face-to-face interviews consisting of demographic factors, socioeconomic status, health care utilization, health care access, health behaviors, and health status. The study protocol was approved by the Charles R. Drew University of Medicine and Science Institutional Review Board (IRB). All participants signed a written informed consent prior to enrollment in the study. Nonrandom sampling was utilized to recruit 620 African-American adults aged 65 years and older. Participants were invited to participate if they met the following criteria: (1) resided in Service Planning Area 6 (SPA 6) in South Los Angeles, (2) identified as African-American or Black, (3) aged 65 years or older, (4) diagnosed and/or managing a cardiometabolic disease, and (5) possess the ability to complete a full interview in English.

### 2.1. Measurements

#### 2.1.1. Flu Vaccination

Vaccination behaviors were assessed by self-report by asking participants to report if they had ever had a flu shot. If the participant answered yes, they responded to the following question, “how long has it been since you had a shot to prevent the flu?” Additionally, participants were asked the following question, “in the past 12 months, has a doctor recommended to you that you should have a flu shot?”

#### 2.1.2. Sociodemographic Covariates

Age, educational attainment, and living arrangements were the covariates in this study. Educational attainment was operationalized as a continuous variable (number of years for school attendance). Higher scores indicated more years of education.

#### 2.1.3. Living Arrangement

We asked our participants whether they lived alone or if there was any other member of the family such as a partner or a spouse who lived with them, which was analyzed categorically as either living alone or living with at least 1 individual.

#### 2.1.4. Financial Strain

Five items were used to measure financial stress of participants. Participants were asked: in the past 12 months, (1) how frequently were you unable to buy the amount of food your family should have? (2) How frequently were you unable to buy the clothes you feel your family should have? (3) How often were you unable to pay your rent or mortgage? (4) How frequently were you unable to pay your monthly bills? (5) How often were you unable to make ends meet? Items were on a 5-level response scale ranging from 1 (never) to 5 (always). A total “financial difficulty” score was calculated, with the average score of five items, ranging from 1 to 5. A high score was indicative of higher financial difficulty. These items are consistent with Pearlin's list of chronic financial difficulties of low Socio-economic status (SES)individuals (*Cronbach* *alpha* = 0.92).

#### 2.1.5. Continuity of Medical Care

Continuity of medical care was measured using 3 items: (1) what type of place do they usually visit to receive medical care (a private doctor's office/private medical group vs. other settings); (2) do they usually go to the same place for medical care; and (3) are they usually attended by the same health provider when they receive medical care. Higher scores indicated more continuity of care.

#### 2.1.6. Satisfaction with Medical Care

Medical care satisfaction was measured using 3 items. Participants reported their level of satisfaction (scale of 1 to 5) with the following: (1) access to, (2) availability of, and (3) quality of medical care. A mean of summary score has a potential range between 1 and 5. A low score indicates less satisfaction.

#### 2.1.7. Disability Status

Disability status was assessed with a single item asking participants whether s/he is disabled (0 = no and 1 = yes).

#### 2.1.8. Self-Rated Health Status (SRH)

This study measured SRH by a single question asking, “In general, would you say your health is 1) Excellent; 2) Very good; 3) Good; 4) Fair; and 5) Poor?” This item is repeatedly used in large-scale national surveys and predicts mortality risk [26].

#### 2.1.9. Major Chronic Conditions and Chronic Obstructive Pulmonary Disease (COPD)

Participants were asked to report whether they have been diagnosed with the following conditions: high blood pressure, diabetes mellitus, heart-related conditions, stroke, and cancer. In addition, participants were asked to report whether they have been diagnosed with asthma or bronchitis.

#### 2.1.10. Depressive Symptoms

The Geriatric Depression Scale was utilized to measure depressive symptoms among this population. This measure has 15 “yes” or “no” items on various symptoms [27]. A summary score was calculated with a range from 0 to 15. A higher score indicated higher levels of depressive symptoms. This tool has high reliability and validity to measure depressive symptoms among older adults both in community and clinical settings [28].

### 2.2. Data Analysis

Our analysis had three parts. The first section was a descriptive analysis of all participants. This descriptive part reported means and standard deviation for continuous measures and frequency and percentages for the categorical variables. Then, we ran both chi-square and independent *t*-test to compare participants who had flu vaccination within last 12 months with those who did not. Additional *t*-test, ANOVA, and chi-squared tests were run to examine correlations between receiving a recommendation for flu vaccination from health care providers and demographic, physical, and mental characteristics of participants. Binary logistic regression was performed to document independent correlates of both flu vaccination within last 12 months and flu vaccination recommended by providers. A *p* value of less than 0.05 was considered significant.

## 3. Results

Table 1 reports the characteristics of the study sample. This study included 620 African-American individuals who were between the ages of 65 and 96 years (*mean* = 74 ± 7.1). Almost 42% of the participants were 75 years of age or older with 65% being composed women. Almost 60% of the sample reported living alone. Twenty-five percent of sample never completed high-school. In regard to health status, only 7% of the sample reported their present health as excellent, while 34% of all participants had fair (29%) or poor health (5%) SRH. The following health illnesses were noted: diabetes mellitus (37%), stroke (15%), and heart-related conditions (30%). Of all respondents, 30% were identified themselves as disabled (Table 1).

### 3.1. Flu Vaccination

Among this same population, 32% (*n* = 194) had never been vaccinated against the flu. Forty-nine percent (*n* = 300) reported being vaccinated within the 12 months prior to the interview (Table 2). Almost 79% of participants indicated that in the past 12 months, their health care provider had told them that “they should have a flu shot.” Of the 478 individuals who indicated that their health care providers recommended flu vaccination, 215 (45%) were not vaccinated within the previous 12 months.

### 3.2. Bivariate Analysis

Table 3 presents the bivariate associations between the flu vaccination and other relevant variables. The following factors were noted to be associated with being less likely to having the flu vaccination within the past 12 months: younger older adults (65–74 years of age), living alone, disability, lower levels of continuity of care, less satisfaction with the accessibility, availability, and quality of medical care, multiple depressive symptoms, and receiving a flu vaccine recommendation from their health care provider. No association between being diagnosed with COPD or other major chronic condition and flu vaccination was detected. In respect to gender, women who participated in this study were 2.08 (CI: 1.36–3.20) times more likely to admit that their provider recommended a flu shot compared to their male counterparts.

### 3.3. Multivariate Logistic Regression

The results of the binary logistic regression examining the correlation between flu vaccination and the independent variables are shown in Table 4. The young-old (65 to 74 years) were 1.54 (CI: 1.07–2.24) times more likely to be vaccinated within the last 12 months compared with the old-old (≥75 years). Participants who lived alone were 0.70 (CI: 0.49–0.98) times less likely to be vaccinated. In addition, participants with a higher level of continuity of care and a higher level of satisfaction with the accessibility, availability, and quality of care were 1.42 (CI: 1.07–1.90) and 1.33 (CI: 1.04–1.69) times more likely to be vaccinated, respectively. Those with a higher number of depression symptoms were 0.92 (CI: 0.84–0.99) times were less likely to be vaccinated. As expected, participants who indicated that their provider had advised them to obtain a flu vaccination were 3.23 (CI: 2.06–5.06) times more likely to be vaccinated. Interestingly, after controlling for other relevant variables, no correlation was detected between self-rated health, being diagnosed with COPD or other major chronic condition and the flu vaccination.

## 4. Discussion

Our study revealed that one out of the three older African-Americans aged 65 years and older residing in South Los Angeles had never received the influenza vaccine. Furthermore, only 49% of participants reported being vaccinated within the 12 months prior to the interview. Compared to the state data from the California Health Interview Survey (CHIS), 36% of individuals aged ≥18 years received an influenza vaccination within the last year. Yet, CHIS data shows African-American were 33% less likely to have been vaccinated than Whites [29]. Moreover, older African-Americans also have lower rates of influenza vaccine uptake in across the nation, including Florida and New York [16, 30]. These findings are consistent in the inequalities in the rate of flu vaccine uptake among underserved older African-Americans, coupled with a disproportionately higher morbidity due to more prevalent and more severe chronic diseases such as cardiovascular (CVD), diabetes, cancer, and COPD places them at extremely high risk for a wide range of undesired health outcomes that include but are not limited to complications, hospitalizations, and premature mortality [22].

One striking finding of our study is the lack of association between self-report of current flu vaccination and being diagnosed with a major chronic condition such as CVD, COPD, diabetes, stroke, and cancer. It has been previously shown that older African-Americans with chronic conditions have lower rates of flu vaccination [7, 31]. A systematic review and meta-analysis of six decades of randomized clinical trials that compared flu vaccine versus placebo among individuals at high risk for CVD showed that the flu vaccine lowered the risk of major adverse cardiovascular events [32]. Further analysis showed no connection between these chronic conditions and being advised by providers to have a flu shot. This could be due to the current ACIP and CDC recommendation for flu shot in all individuals unless contraindications exist. This finding, however, should be further investigated in future research.

National data clearly show that having a regular place of care and a regular care provider may be associated with higher chance of receiving clinical preventive services among older adults [33]. Systematic reviews of recent studies show that one of the major determinants of influenza vaccination among older adults is a recommendation by their health care provider [34]. Indeed, there are strong evidences that providers may have a profound influence on individuals' intentions to receive vaccination; thus, physicians have a unique role in promoting influenza vaccination rates at a national level [35]. Along those lines, it has consistently been documented that flu recommendation by primary care providers is an influential factor for immunization among older adults [36]. Therefore, it is necessary that providers of older African-Americans educate this group on side effects, vaccine safety, and drug interactions [13, 37].

Older adults whose usual physician is a primary care generalist have higher chance of immunization than those who receive care from specialist (usual) physician [38, 39]. Older adults who were more satisfied with the information-giving skills and accessibility of their physicians had higher immunization rates than their counterparts [38]. Still, older White adults who have regular health care providers are more likely to have higher influenza vaccine uptake [6]. Among minority populations, complacency and lack of confidence in vaccine efficacy impact vaccine behaviors. This may be attributed to being less likely to receive educational advice from providers about the effectiveness of vaccination amongst minority population [5, 22]. Consistent with these findings, our data confirmed that both “continuity of care” and “satisfaction with access, availability, and quality of medical care” strongly associated with current flu vaccinations. Knowing that African-Americans have lower levels of knowledge about influenza and influenza vaccine, primary health care providers can enhance uptake by addressing key gaps such as how the vaccine itself works, its effectiveness, and myths about the vaccine causing influenza [22]. Africans-Americans, recognized as having a high rate of church attendance, may not take the vaccine if they encounter negative influenza attitudes within the church [40]. These findings together highlight the crucial role of having a usual source of care as a gatekeeper in locations that are prevalent with African-Americans (i.e., churches, barbershops, and beauty salons) to facilitate and implement preventive medical care among underserved minority older adults.

Our data shows that living arrangement is associated with decreased vaccination among our sample of underserved African-American older adults. Examination of flu vaccine behavior among high-risk African-American and Whites shows that African-American high-risk adults are less likely to be immunized than White counterparts [41]. Consistent with our study, a recent systematic review documented higher seasonal influenza vaccine uptake (OR = 1.39 and CI: 1.16–1.68) among individuals not living alone [42]. Social isolation, loneliness, and living also are overlooked factors in health care delivery among older vulnerable adults. A meta-analysis of three decades studies conducted on social isolation and loneliness as risk factors of risk of mortality shows that living alone significantly increased likelihood of mortality [43]. Quantification of associations between living alone and lower vaccine uptake should enable health professionals target specific underserved African-American older adults to tackle vaccine-related inequalities.

There is conflicting data on potential impact of depression on preventive care. A European study showed that older adults with depressive symptoms are frequent users of health care but not preventive services [44]. However, the Canadian National Population Health Survey revealed no effect of major depression on some preventive behaviors in either cross-sectional or longitudinal analyses [45]. Results of the National Behavioral Risk Factor Surveillance System (BRFSS) data United States show a significant role of health-related quality of life and social determinants (SDOH) on flu vaccine among adults 18 years of age and older. This national study shows that participants with poor mental health had 19% lower odds of receiving flu vaccine in comparison to their counterparts. In comparison to individuals without depressive disorder, participants with depression had 1.29 (95% CI: 1.21-1.38) times higher odds of receiving flu vaccine [46]. We documented that among our sample of underserved African-American older adults, participants with a higher number of depression symptoms were less likely to be vaccinated. An additional study exhibited that depression reduces adherence to drug regimens [47]. As depression is more chronic/disabling and less likely to be treated in African-American [48, 49], there is a need to screen and treat depression as a strategy to enhance preventive care management of African-American older adults. Literature also shows that integrating depression treatment into care for physical chronic conditions significantly increases adherence to drug regimens [50].

Study implications may be applicable among local community organizations for the implementation of evidence-based programs that increase education on influenza education [15]. Systems that have evidence of success are client reminder and recall programs, clinic-based client centered education, provider education and participation, and home health and other incentivized interventions [15]. Additionally, there is an indication for more information about how the vaccine works to prevent influenza infection and an approach for public health to deliver targeted messaging that interest older adults [15]. Influenza-related illnesses and deaths along with hospitalizations related to influenza are also important in messaging especially for those persons suffering from chronic diseases [15].

Several factors limiting this study must be noted. First, generalizability of the results is limited given that we used consecutive sampling. Due to the cross-sectional design of our study, we cannot make causal inference from our associations. Second, we did not have access to objective medical histories and records of the participants; therefore, we relied on self-report of vaccination, health status, and physical and chronic conditions.

## Figures and Tables

**Table 1:** Characteristics of participants (*n* = 620).

	*n*	%
Gender		
Male	215	34.7
Female	405	65.3
Age		
65-74	360	58.1
≥75	260	41.9
Education		
No high school diploma	156	25.2
High school diploma	217	35.0
Some college or college degree	247	39.8
Living alone		
No	249	40.2
Yes	371	59.8
Disability status		
No	433	69.8
Yes	187	30.2
Chronic obstructive pulmonary disease		
No	468	75.7
Yes	150	24.3
		Mean ± SD
Age (years: 65-96)		74.0 ± 7.02
Education attainment (1–16)		12.7 ± 2.3
Financial strains (1–5)		1.6 ± 0.99
Continuity of care (0: low–3: high)		2.6 ± 0.61
Satisfaction with access, availability, and quality of medical care (1: low to 5: high)		3.4 ± 0.73
Self-rated health status (1: excellent to 5: poor)		3.1 ± 0.99
Depression (GDS: 0-15)		2.1 ± 2.44
Major chronic conditions (1–5)		2.1 ± 0.86

**Table 2:** Flu vaccination and providers' recommendation (*n* = 620).

	*n*	%
Flu vaccination recommended by providers		
No	129	21.3
Yes	478	78.7
Flu vaccination within last 12 months		
No	310	50.8
Yes	300	49.2
History of flu vaccination		
Never had vaccination before	194	31.8
At least once (but not in last 12 months)	116	19.0
Vaccinated within last 12 months	300	49.2

**Table 3:** Bivariate correlates of flu vaccination within last 12 months (*n* = 620).

Independent variables	Flu vaccination (last 12 months)		*p*
	No *n* (%) or (*X* ± SD)	Yes *n* (%) or (*X* ± SD)	
Gender			0.967
Male	108 (51)	105 (49)	
Female	202 (51)	195 (49)	
Age			0.010
65-74	196 (55)	159 (45)	
≥75	114 (45)	141 (55)	
Education (scale: 1–15)	(12.6 ± 2.38)	(12.74 ± 2.39)	0.517
Living alone			0.039
No	112 (46)	133 (54)	
Yes	198 (54)	167 (46)	
Disabled			0.006
No	201 (47)	225 (53)	
Yes	109 (59)	75 (41)	
Financial stress (scale: 1-5)	1.70 ± 1.01	1.54 ± 0.92	0.491
Continuity of care (scale: 0–3)	2.51 ± 0.65	2.6 ± 0.56	0.026
Satisfaction with access, availability, and quality of medical care (scale: 0–5)	3.31 ± 0.71	3.50 ± 0.77	0.020
Self-rated health status (scale: 1–5)	3.04 ± 1.01	3.05 ± 0.97	0.954
Chronic obstructive pulmonary disease			0.993
No	235 (51)	226 (49)	
Yes	75 (51)	72 (49)	
Major chronic conditions (scale: 1–5)	2.05 ± 0.88	2.1 ± 0.84	0.281
Depression symptoms (scale: 1-15)	2.39 ± 2.64	1.77 ± 2.16	0.002
Flu vaccination recommended			0.001
No	94 (73)	35 (27)	
Yes	215 (45)	263 (55)	

**Table 4:** Binary logistic regression between independent variables and flu vaccination (*n* = 620).

Independent variables	Flu vaccination within last 12 months		Sig.
	OR	95% CI	
Gender			
Male	0.77	0.54–1.12	0.175
Female	1.00		
Age			
65-74	1.54	1.07–2.24	0.019
≥75	1.00		
Education (scale: 1–15)	1.03	0.95–1.11	0.563
Living alone			
No	0.70	0.49–0.98	0.049
Yes	1.00		
Disabled			
No	0.70	0.47–1.02	0.070
Yes	1.00		
Financial stress (scale of 1: low to 5: high)	1.02	0.84–1.24	0.856
Continuity of care (scale 0: low to 3: high)	1.42	1.07–1.90	0.017
Satisfaction with access, availability, and quality of medical care (scale of 1: low to 3: high)	1.33	1.04–1.69	0.022
Self-rated health status (scale of 1: excellent to 5: poor)	1.14 1.00	0.95–1.37	0.162
Chronic obstructive pulmonary disease			
No	1.20	0.79–1.82	0.383
Yes	1.00		
Number of major chronic conditions (scale: 1–5)	1.12 1.00	0.91–1.37	0.275
Depression symptoms (scale: 1-15)	0.92 1.00	0.84 – 0.99	0.041
Provider recommended flu vaccination			
No	3.23	2.06–5.06	0.0001
Yes	1.00		

Notes: -2 log likelihood: 767.8; Sig: 0.0001; Nagelkerke: 0.144.

## Data Availability

Data is available upon request.
